# THE USE OF QUALITATIVE METHODS IN UNDERSTANDING FEARS FOR THE FUTURE AMONG AGING CAREGIVERS OF ADULTS WITH ASD

**DOI:** 10.1093/geroni/igz038.2043

**Published:** 2019-11-08

**Authors:** Christina N Marsack-Topolewski, Christina Topolewski, Jillian Graves

**Affiliations:** Eastern Michigan University, Ypsilanti, Michigan, United States

## Abstract

Understanding the realities of enduring caregivers as they age is essential to providing adequate supports and services to support the both caregiver and care recipient. An analysis of 51 one-on-one interviews was used to examine the lived experiences of aging caregivers, specifically regarding future care plans and barriers for their adult children diagnosed with autism spectrum disorders (ASD). Using a phenomenological research design, our qualitative analysis included line-by-line analysis, followed by PI and co-investigator collaboration to discuss findings. As a collaborative process, the codes and themes were refined by collapsing and consolidating codes. Intensive interviewing, coupled with systematic qualitative analysis, yielded substantive responses around aging caregivers’ experiences. Results highlighted four main themes that emerged from the interview responses, including (1) difficulty identifying caregiving support for the future, (2) barriers to making plans/decisions, (3) fear of the unknown, and (4) feeling the need to make plans and decisions now.

